# Supplementary material for: Effect of Different Clarification Treatments on the Volatile Composition and Aromatic Attributes of ‘Italian Riesling’ Icewine
Source: Molecules. 2020 Jun 8;25(11):2657. doi: 10.3390/molecules25112657 (PMC7321132; doi:10.3390/molecules25112657)
Supplement: Supplementary file 1 [file molecules-25-02657-s001.pdf]

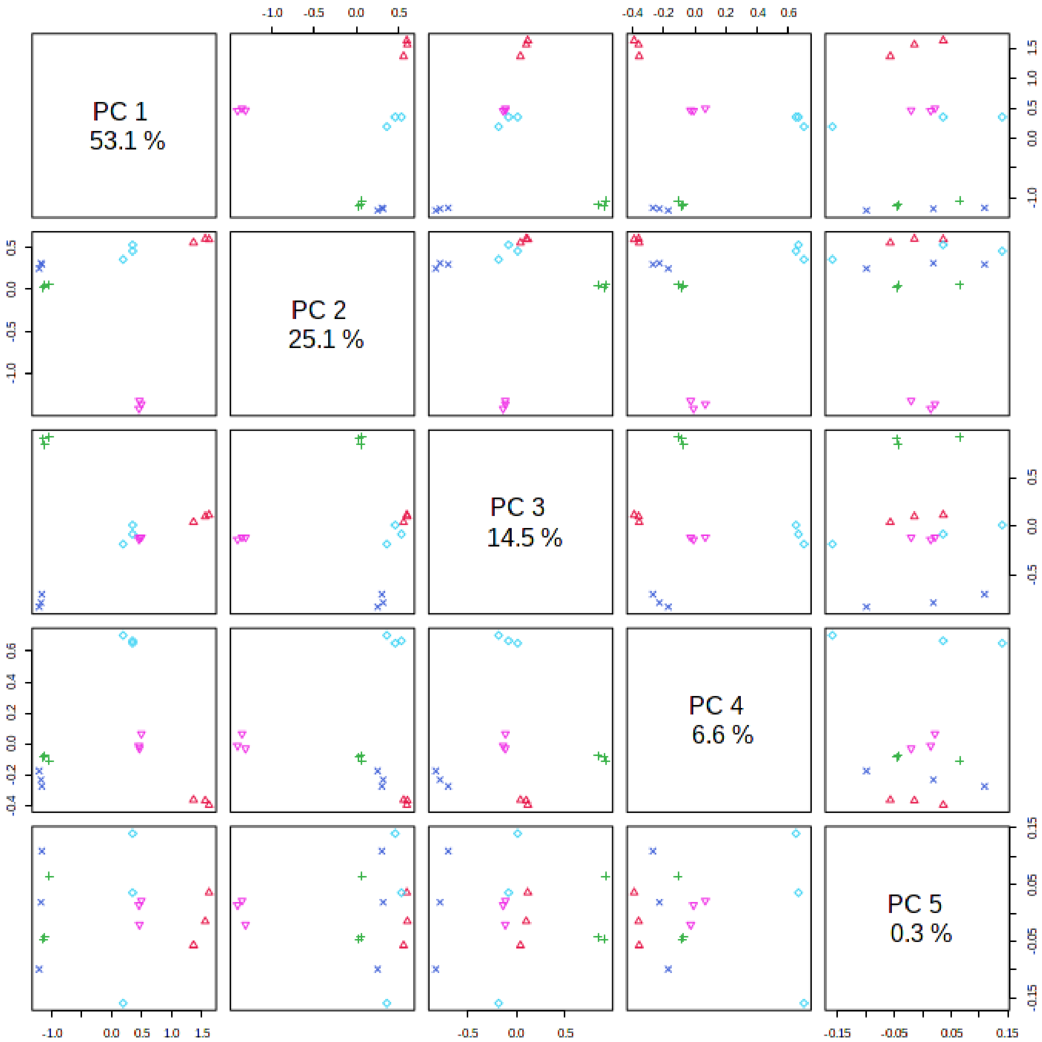

Figure S1: Principal component analysis score plot of volatile compounds (OAV>1) in wine samples with different clarification treatments

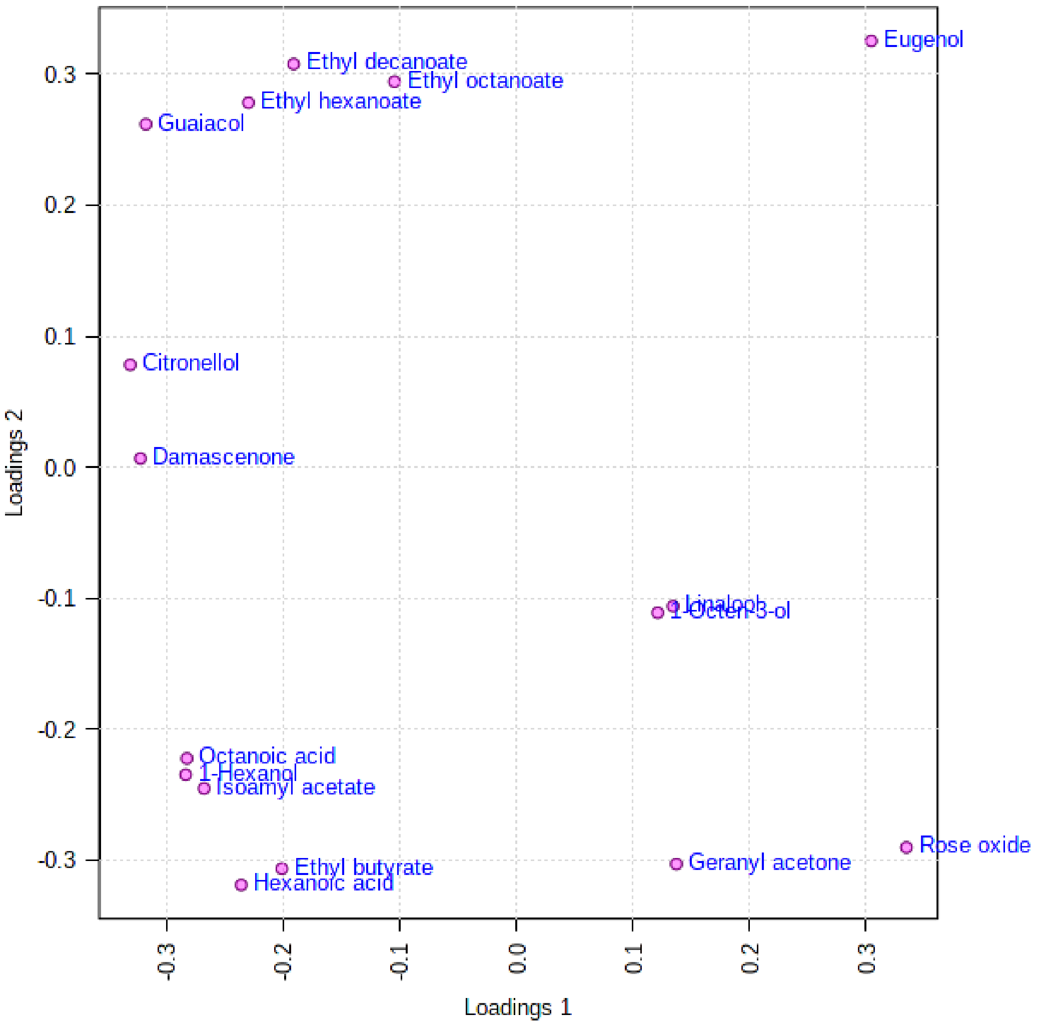

Figure S2: Principal component analysis loading plot of volatile compounds (OAV>1) in wine samples with different clarification treatments

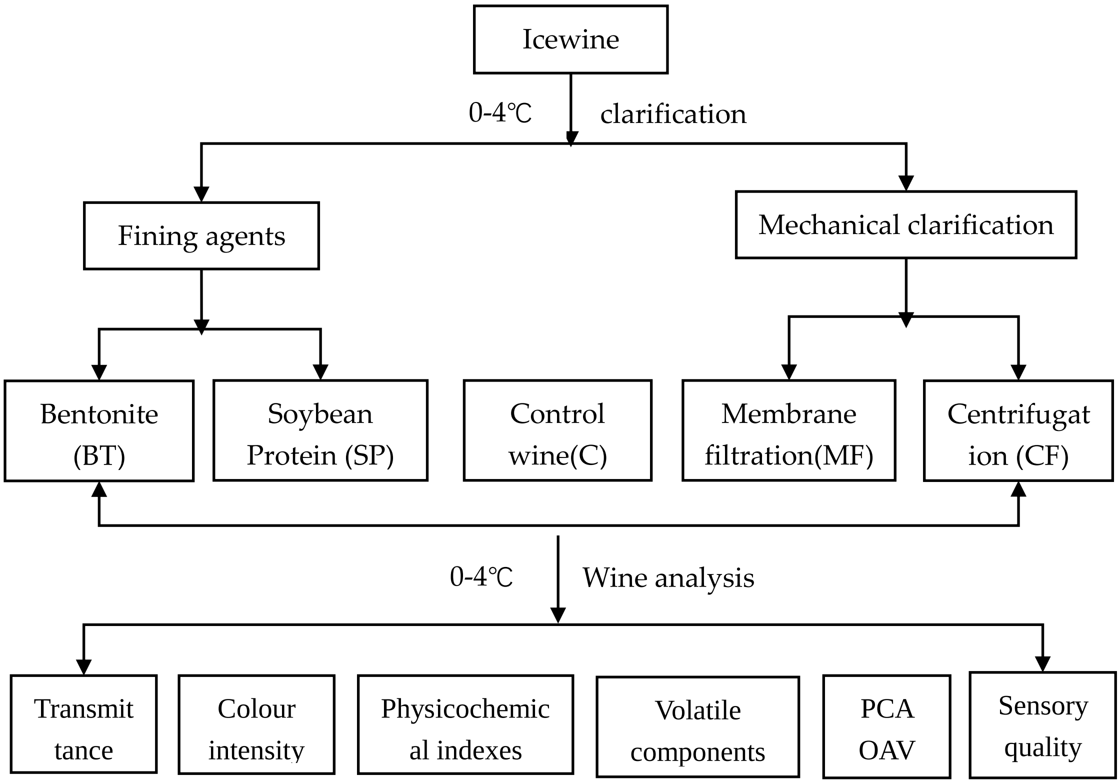

Figure S3: The diagram of experiment
